# Comparison of probiotics to lactulose for minimal hepatic encephalopathy in patients with cirrhosis: a meta-analysis of randomized controlled trials

**DOI:** 10.3389/fmed.2026.1780891

**Published:** 2026-02-12

**Authors:** Qiufeng He, Zhili Chen, Yang Deng, Chuangjie Mao, Yue Fan

**Affiliations:** 1 Department of Hepatology, Public Health Clinical Center of Chengdu, Chengdu, Sichuan, China; 2 Department of Blood Transfusion, Deyang People’s Hospital, Deyang, Sichuan, China

**Keywords:** adverse events, lactulose, minimal hepatic encephalopathy, probiotics, randomized controlled trials

## Abstract

**Background:**

Minimal hepatic encephalopathy (MHE) represents a reversible, early-stage form of hepatic encephalopathy (HE). Although probiotics have been extensively studied for MHE management, direct comparative evidence against standard lactulose therapy remains limited. This meta-analysis aimed to quantitatively evaluate the relative efficacy and safety of probiotics versus lactulose in cirrhotic patients with MHE.

**Methods:**

PubMed, the Cochrane Library, Embase, Web of Science, and the Chinese Biomedical Literature Database (CBM) were systematically searched from inception to August 2025 to identify randomized controlled trials (RCTs) comparing probiotics with lactulose for the treatment of MHE in patients with cirrhosis. Extracted outcomes included MHE reversal, overt hepatic encephalopathy (OHE) development, serum ammonia reduction, and adverse events (AEs).

**Results:**

Five studies involving 345 cirrhotic patients were included. Pooled analyses showed no statistically significant differences between probiotics and lactulose in reversing MHE (RR: 0.98, 95% CI: 0.79–1.20; *p* = 0.822), preventing OHE development (RR: 1.40, 95% CI: 0.75–2.61; *p* = 0.289), and reducing serum ammonia levels (SMD: −0.05, 95% CI: −0.29 to 0.19; *p* = 0.678). In contrast, probiotics were associated with a significantly lower incidence of AEs compared with lactulose (RR: 0.17, 95% CI: 0.05–0.60; *p* = 0.005).

**Conclusion:**

This meta-analysis found no evidence that probiotics are superior or inferior to lactulose in terms of MHE reversal, prevention of OHE, and reduction of serum ammonia levels. Probiotics were associated with fewer AEs, suggesting a potential safety advantage. Further large-scale, high-quality RCTs are warranted to confirm these findings.

## 1 Introduction

Hepatic encephalopathy (HE) is a multifaceted and potentially reversible neuropsychiatric syndrome that commonly occurs in patients with liver cirrhosis, manifesting across a clinical spectrum from minimal hepatic encephalopathy (MHE) to overt hepatic encephalopathy (OHE), the latter representing the most severe form characterized by altered consciousness (1, 2). MHE represents the earliest stage of HE, presenting with subtle, nonspecific cognitive deficits detectable only through specialized neuropsychological and psychometric tests (3, 4). Its prevalence exhibits considerable geographical variation, influenced by differing diagnostic criteria and population characteristics. In patients with cirrhosis, the global incidence of MHE ranges from 30% to 84% (5–7). MHE adversely impacts cognitive and motor functions, leading to attention deficits, fatigue, sleep disturbances, impaired driving capacity, and reduced work productivity (8, 9). These impairments diminish health-related quality of life (HRQL) and escalate socioeconomic costs via accidents and productivity losses (10). Critically, MHE predicts progression to OHE and independently increases mortality risk in cirrhosis, reflecting its role as a precursor to severe complications (11, 12).

The pathogenesis of MHE remains incompletely elucidated but aligns with established neurotoxic mechanisms of HE. Hyperammonemia plays a central role and is further exacerbated by intestinal dysbiosis, oxidative stress, infection, and increased intestinal permeability (13, 14). Targeted pharmacologic interventions for MHE include lactulose, rifaximin, L-ornithine L-aspartate (LOLA), probiotics, and branched-chain amino acids (BCAAs). Among these, lactulose is recommended as first-line therapy by clinical guidelines and expert consensus (1, 2, 15, 16). However, its frequent gastrointestinal adverse effects (AE), such as diarrhea and flatulence, often compromise patient adherence.

Probiotics have emerged as a potential alternative strategy, acting through modulation of gut microbiota composition, suppression of pathogenic bacteria, enhancement of intestinal barrier integrity, and attenuation of systemic inflammation and oxidative stress (17, 18). While these agents are widely discussed in prior meta-analyses, those studies often combined direct evidence with indirect comparisons or non-randomized data, which can limit the certainty of conclusions about their relative merits specifically in direct comparisons. To address this evidence gap, we conducted a meta-analysis restricted to randomized controlled trials (RCTs) directly comparing probiotics with lactulose in patients with cirrhosis and MHE. This study aims to explore the comparative efficacy and safety of probiotics versus lactulose, providing preliminary insights to help inform clinical considerations and highlight areas for future investigation.

## 2 Materials and methods

### 2.1 Study protocol and registration

This meta-analysis was conducted in strict adherence to the Preferred Reporting Items for Systematic Reviews and Meta-Analyses (PRISMA 2020) guidelines (19), and the completed PRISMA 2020 Checklist is provided as Supplementary File 1. A detailed study protocol was developed *a priori* to guide all aspects of the research process. To ensure transparency and reproducibility, the finalized version of this protocol is available as Supplementary File 2.

### 2.2 Eligibility criteria

The search followed the following PICO framework:

Population (P): Adult (≥18 years) patients with cirrhosis diagnosed with MHE.

Intervention (I): Probiotic-based therapy, including single- or multi-strain probiotics, synbiotics, and prebiotics.

Comparator (C): Lactulose.

Outcome (O): Primary outcome: MHE reversal; Secondary outcomes: development OHE, reduction in serum ammonia levels, and the incidence of AEs.

Inclusion criteria: (1) cirrhotic patients diagnosed with MHE; (2) adult patients (age ≥18 years); (3) RCTs comparing probiotics with lactulose; (4) availability of sufficient data for at least one pre-specified outcome.

Exclusion criteria: (1) studies involving patients with OHE at baseline; (2) pediatric participants (age <18 years); (3) incomplete or unavailable outcome data; (4) non-human studies; (5) uncontrolled or nonrandomized trials.

### 2.3 Search strategy

The systematic search was performed from inception to August 2025 across five electronic databases: PubMed, the Cochrane Library, Embase, Web of Science, and China Biomedical Literature Database (CBM), with no language restrictions. To maximize retrieval sensitivity, search strategies combined Medical Subject Headings (MeSH) with relevant free-text terms for the key concepts of: “minimal hepatic encephalopathy”, “cirrhosis”, “probiotics”, “lactulose”, and “randomized controlled trials”. In addition, reference lists of relevant reviews and included studies were manually screened to identify additional eligible trials. The complete search strategies for all databases are provided in Supplementary File 3 to enhance reproducibility.

### 2.4 Study selection

All identified records were imported into EndNote X7 (Thomson Reuters, New York, United States), where duplicate citations were automatically removed using the software’s built-in function. The remaining articles were then independently screened in duplicate by two reviewers (QFH and ZLC) at two sequential stages: first by title and abstract, and subsequently by full text. Any disagreements during the screening process were resolved through discussion or, if necessary, by consultation with a third, senior reviewer (YF or CJM). Animal studies and publications without accessible full texts were excluded during full-text screening.

### 2.5 Data extraction

Data extraction was independently performed by QH and ZC using a standardized form, including publication year, country, sample size, demographic characteristics, intervention details (strain, dosage, duration), and MHE diagnostic methods. The primary outcome was MHE reversal, while secondary outcomes included development of OHE, serum ammonia reduction, and incidence of AEs. Inter-rater reliability for key extracted variables was evaluated using Cohen’s Kappa coefficient, and any discrepancies were resolved through discussion or consultation with a third reviewer (YF or CM).

### 2.6 Quality assessment

The methodological quality of included RCTs was assessed using the Cochrane Risk of Bias 2 (RoB 2) tool (20), evaluating five domains: randomization process, deviations from intended interventions, missing outcome data, outcome measurement, and selection of reported results. Each domain was judged as “low risk,” “some concerns” or “high risk.” Two reviewers (QH and ZC) independently conducted the assessments, with disagreements resolved by discussion or consultation with a third reviewer (YF or CM).

### 2.7 Statistical analysis

All statistical analyses were performed using Stata version 12.0. For dichotomous outcomes, risk ratios (RRs) with 95% confidence intervals (CIs) were calculated, while standardized mean differences (SMDs) with 95% Cis were used for continuous outcomes. Statistical heterogeneity was assessed using the I^2^ statistic and Cochran’s Q test. Given the small number of included studies, heterogeneity estimates were interpreted with caution, as I^2^ values may be unreliable in meta-analyses with limited sample size. Considering the expected clinical heterogeneity among included studies (e.g., probiotic strains, dosages, and treatment duration), random-effects models were used as the primary analytical approach. Random-effects models account for both within-study and between-study variance, providing more conservative effect estimates when heterogeneity is anticipated. Fixed-effect models were additionally performed as sensitivity analyses to assess the robustness of pooled results. Publication bias was explored using funnel plots and Egger’s regression test (21); however, due to the limited number of included studies, these analyses were considered exploratory, and no definitive conclusions regarding publication bias were drawn. All statistical tests were two-tailed, with *p* < 0.05 considered statistically significant.

## 3 Results

### 3.1 Literature selection and study characteristics

**Figure 1:**
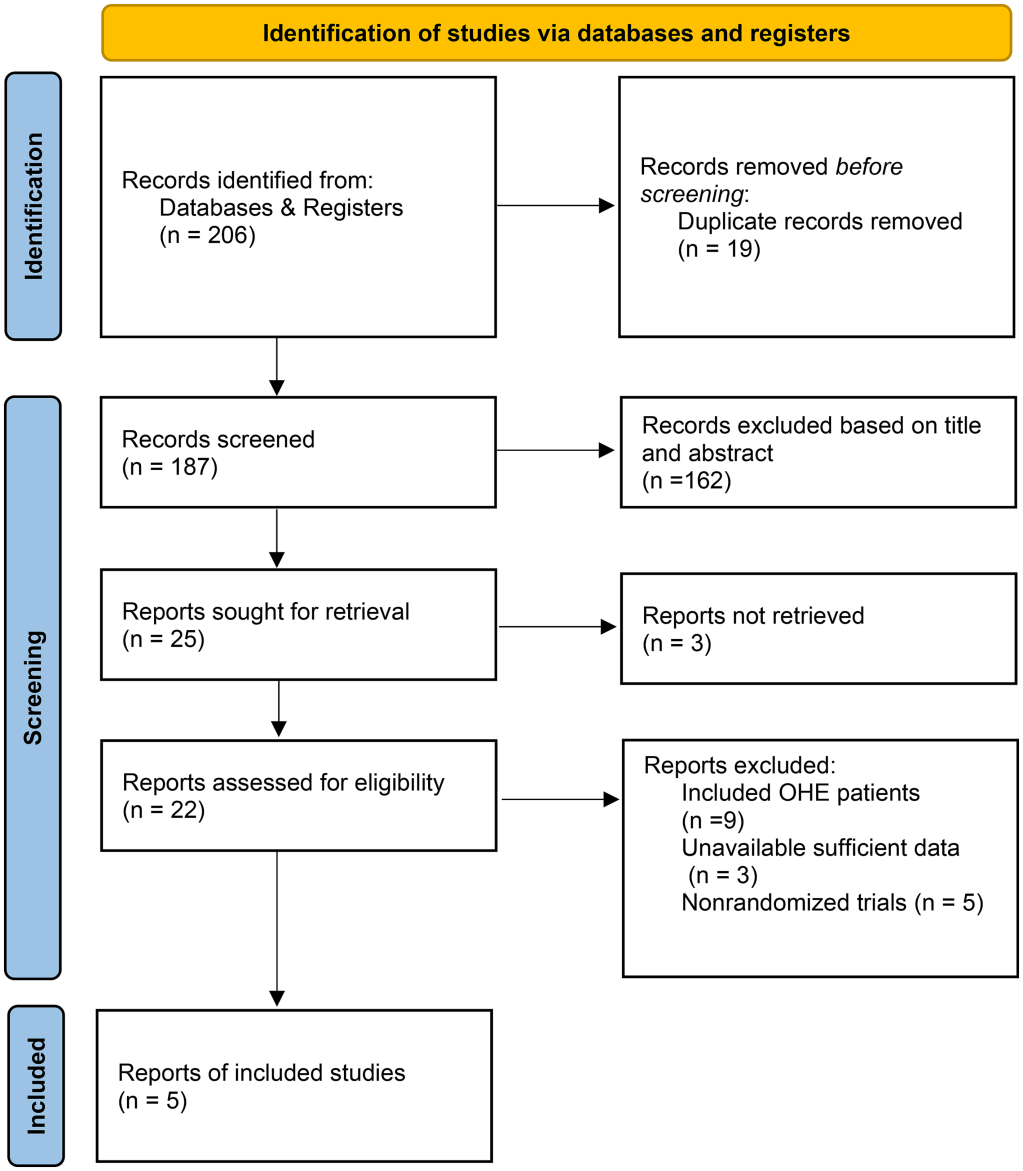
PRISMA flow diagram of the literature search and study selection process.

The initial search yielded 206 publications. After removing 19 duplicates and excluding 162 studies during title and abstract screening, 25 underwent full-text evaluation. Seventeen studies were excluded based on predefined criteria, and three lacked sufficient outcome data, ultimately leaving five RCTs (22–26) for analysis (Figure 1). The final analysis comprised 345 participants, of which 170 (49.3%) participants were assigned to the probiotics group. Studies spanned 2008–2015 and enrolled predominantly 40–50 years old cirrhotic patients from India, Egypt, and China.

Lactulose dosing was standardized (30–60 mL/day), while probiotic formulations and doses varied across trials. MHE diagnostic methods were explicitly reported in all studies. Key baseline characteristics are detailed in Table 1. Cohen’s kappa coefficient for data extraction was 0.86, indicating strong agreement between reviewers.

**Table 1:** Basic characteristics of the included studies.

Study	Year	Country	Treatment duration	Tests used for diagnosis of MHE	No. of patients		Age		Sex (Male/Female)	
					Probiotics	Lactulose	Probiotics	Lactulose	Probiotics	Lactulose
Sharma et al. (22)	2008	India	1 month	NCT, FCT, P300ERP	31	31	43.5 ± 12.1	39.5 ± 13.0	NA	NA
Mittal et al. (23)	2011	India	3 months	NCT, FCT, BDT	40	40	44.25 ± 11.8	43.85 ± 10.9	30/10	32/8
Ziada et al. (24)	2013	Egypt	1 month	NCT, DST, SDT	26	24	50.3 ± 7.8	48.8 ± 8.2	19/7	18/6
Zhao et al. (25)	2013	China	1 month	NCT, DST	40	40	44.25 ± 11.85	43.85 ± 11.10	30/10	32/8
Pratap et al. (26)	2015	India	2 months	NCT, FCT, P300ERP	33	40	39.6 ± 11.4	44.2 ± 10.4	NA	NA

MHE, Minimal hepatic encephalopathy; NCT, number connection test; FCT, figure connection test; P300ERP, P300 auditory event-related potential; BDT, block design test; DST, digit symbol test; SDT, serial-dotting test; NA, not available.

### 3.2 Quality assessment

According to the Cochrane risk of bias tool, random sequence generation was adequately described in most studies, though allocation concealment methods were reported sufficiently in only approximately half. Due to predominantly open-label designs, awareness of interventions among participants and personnel created some risk of performance bias. However, objectivity of primary outcomes generally resulted in low-to-moderate detection risk. One study fully adhered to protocol-specified outcomes, others lacked sufficient detail on pre-specified analysis plans. In summary, the methodological quality of the included studies was considered moderate risk of bias profile, with the main limitation being the lack of adequate blinding. Supplementary Figure S1 present details of the risk of bias assessments for individual studies (see Table 2).

**Table 2:** Types and doses of probiotics in each included study.

Study	Types of probiotics	Doses
Sharma et al. (22)	*streptococcus faecalis*, *clostridium butyricum*, bacillus mesentricus, lactic acid bacillus	1 capsule, 3 times per day
Mittal et al. (23)	110 billion colony forming units bd	1 capsule, 2 times per day
Ziada et al. (24)	106 *L. acidophilus*	1 capsule, 3 times per day
Zhao et al. (25)	110 billion colony forming units bd	1 capsule, 2 times per day
Pratap et al. (26)	*Streptococcus thermophilus*, *Bifidobacterium breve*, *Bifidobacterium longum*, *Bifidobacterium infantis*, *Lactobacillus acidophilus*, *Lactobacillus plantarum*, *Lactobacillus paracasei*, *Lactobacillus bulgaricus*	2 capsules, 2 times per day

### 3.3 MHE reversal

**Figure 2:**
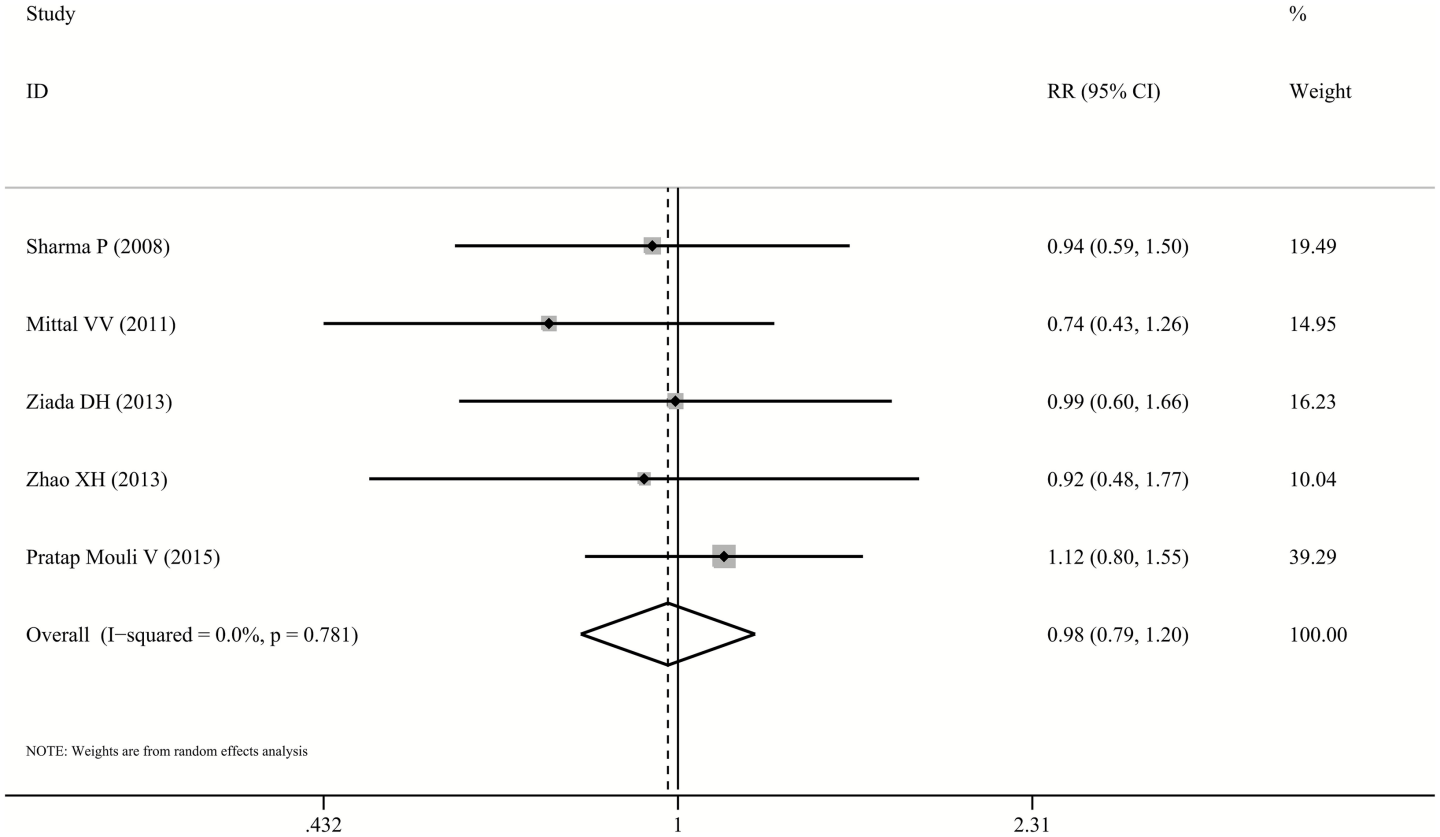
Forest plot for MHE reversal.

Data on MHE reversal outcomes were available from all five studies comparing probiotics with lactulose. Across included trials, MHE reversal was achieved in 30.0%–69.7% of probiotic patients and 32.5%–62.5% of lactulose patients. The pooled random-effects model demonstrated no statistically significant difference between probiotics and lactulose in MHE reversal (RR: 0.98, 95% CI: 0.79–1.2, *p* = 0.822, I^2^ = 0.0%; Figure 2), indicating no evidence of superiority or inferiority between the two interventions.

### 3.4 OHE development

**Figure 3:**
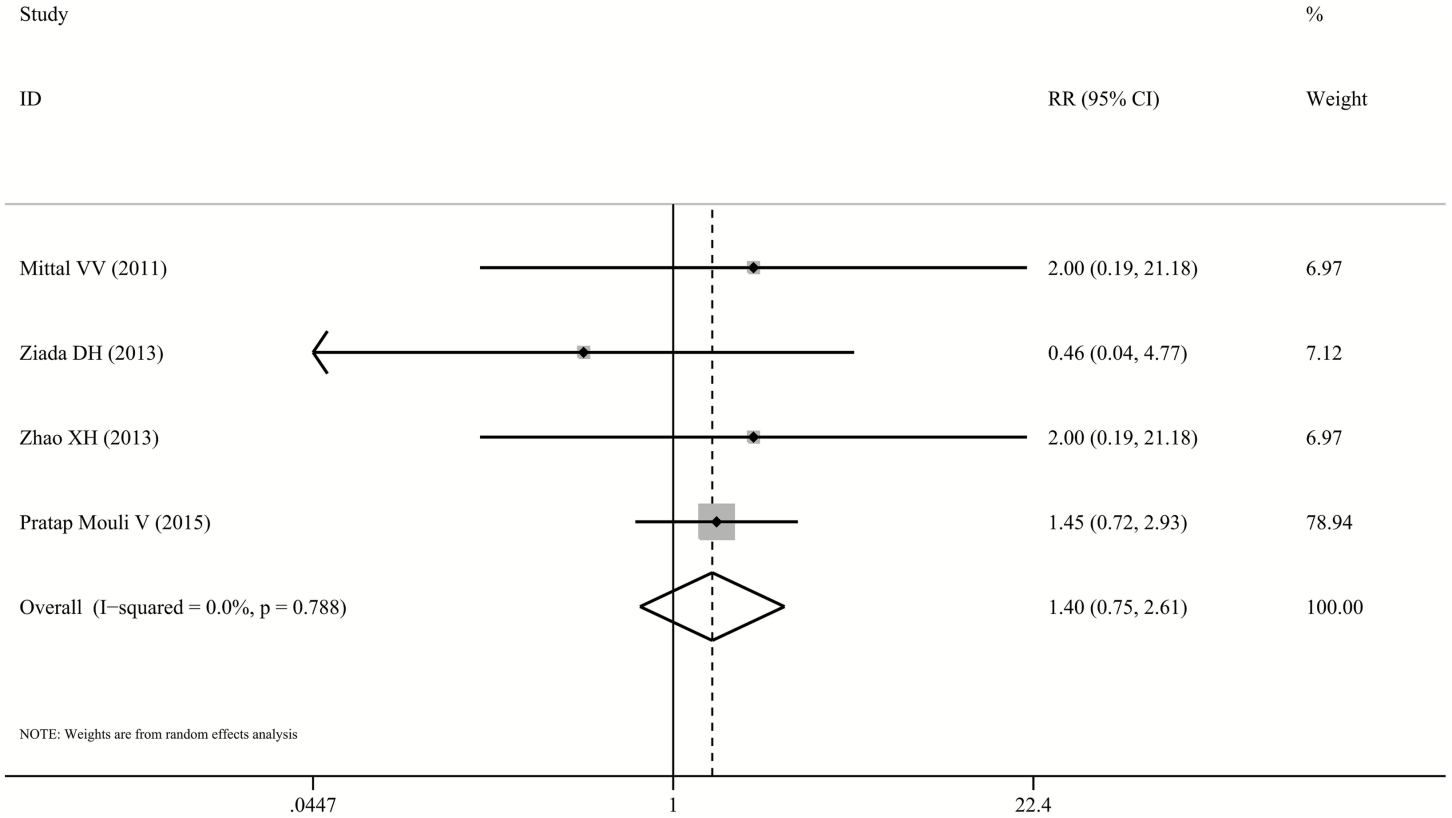
Forest plot for OHE development.

The outcome of OHE development was reported in four included trials (23–26), with incidence rates varying between 3.8%–36.4% for probiotics and 2.5%–25.0% for lactulose. Pooled analysis showed that the risk of developing OHE did not differ significantly between patients receiving probiotics and those receiving lactulose (RR: 1.40, 95% CI: 0.75–2.61, *p* = 0.289, I^2^ = 0.0%; Figure 3).

### 3.5 Serum ammonia reduction

**Figure 4:**
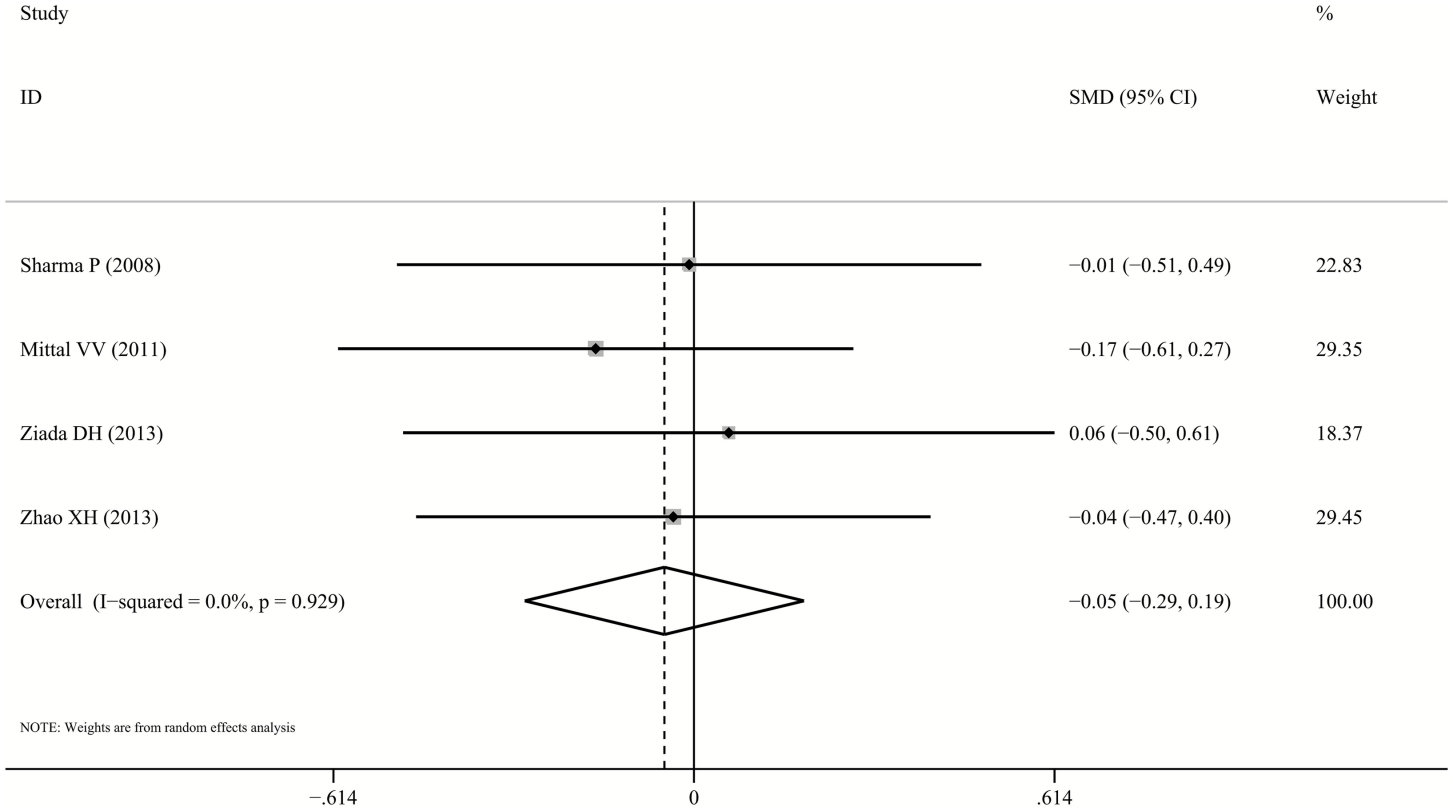
Forest plot for ammonia reduction.

One trial (26) failed to provide serum ammonia data, but both probiotics and lactulose significantly reduced levels from baseline. Pooled analysis of remaining four studies demonstrated no significant difference between probiotics and lactulose in reducing serum ammonia levels (SMD: -0.05, 95% CI: −0.29–0.19, *p* = 0.678, I^2^ = 0.0%; Figure 4).

### 3.6 Treatment safety

**Figure 5:**
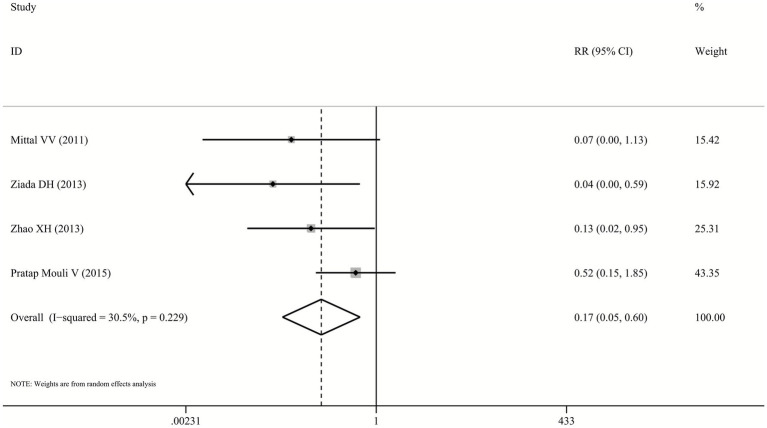
Forest plot for the incidence of AEs.

The risk of AEs was significantly lower in the probiotics group compared to the lactulose group (RR: 0.17, 95% CI: 0.05–0.6, *p* = 0.005, I^2^ = 30.5%; Figure 5), based on data from four studies (23–26). However, several included trials reported only the total number of patients experiencing any AE without specifying the types, incidence rates of individual AEs, or severity grading. Therefore, detailed stratified analysis of specific AE types and severity levels was not feasible. Commonly reported AEs included nausea, diarrhea, and abdominal pain. No serious AEs or significant laboratory abnormalities were documented in the included studies.

### 3.7 Sensitivity analysis

A sensitivity analysis for both primary and secondary outcomes was conducted to assess the robustness of the findings. By excluding studies considered at higher risk of bias according to the Cochrane Collaboration risk assessment tool, we identified consistency of findings and no statistically significant changes were noted for all the comparisons performed. The sensitivity analyses by the fixed-effects mode yielded similar results to the random-effects model (Supplementary Figures S2–S4).

### 3.8 Publication bias

Publication bias for the primary outcome of MHE reversal was explored using funnel plots and Egger’s regression test as part of an overall assessment of the robustness of the evidence. Given the limited number of included studies, publication bias analyses were interpreted cautiously.

**Figure 6:**
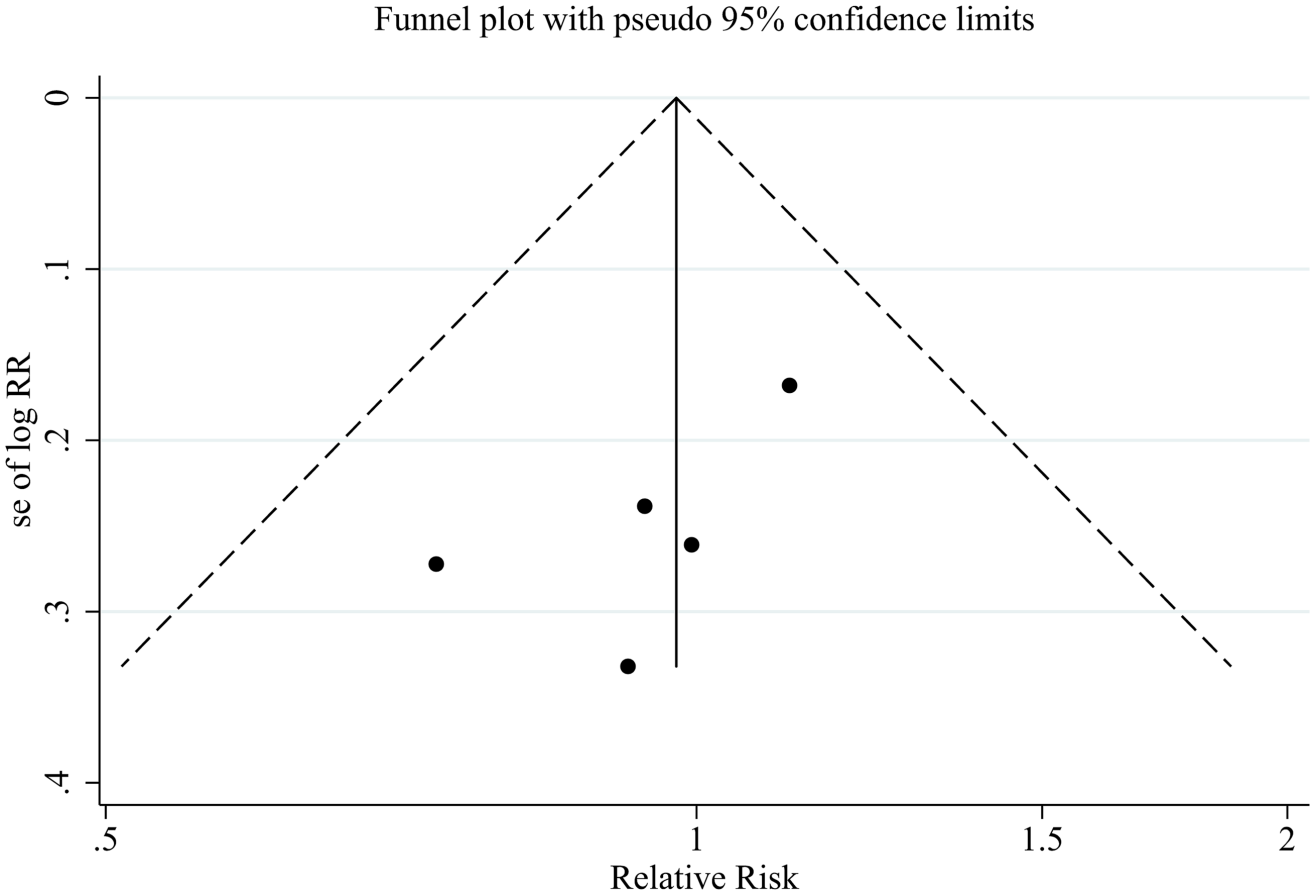
Funnel plot assessing publication bias for the primary outcome.

The funnel plot appeared approximately symmetrical (Figure 6), and Egger’s test did not indicate statistically significant small-study effects (*p* = 0.162). However, due to insufficient statistical power, these findings cannot exclude the possibility of publication bias.

## 4 Discussion

This meta-analysis systematically evaluated and compared the efficacy and safety of probiotics and lactulose for the management of MHE in patients with cirrhosis. Based on data from five RCTs, no statistically significant differences were observed between the two interventions with respect to MHE reversal, prevention of progression to OHE, or reduction of serum ammonia levels. These findings should be interpreted as indicating no evidence of superiority or inferiority, rather than definitive equivalence, particularly given the limited number of studies and the absence of equivalence or non-inferiority trial designs. The relatively wide confidence intervals and modest sample sizes further constrain the precision of effect estimates. Although probiotics were associated with a lower incidence of reported AEs, this finding should be interpreted with caution due to heterogeneity and the potential for reporting bias. Overall, probiotics may represent a potential therapeutic alternative for selected patients, particularly those who are intolerant to lactulose.

Although the pathogenesis of hepatic encephalopathy is multifactorial, hyperammonemia is widely recognized as a central contributor to the development of MHE. In cirrhosis, impaired ammonia metabolism, intestinal dysbiosis, increased intestinal permeability, and luminal alkalization collectively promote excessive ammonia production and absorption (27, 28). In parallel, cirrhosis-associated neuroinflammation compromises blood–brain barrier integrity, increasing central nervous system vulnerability to ammonia toxicity (29). Lactulose remains the guideline-recommended first-line therapy for HE, primarily due to its ammonia-lowering effects. Through colonic bacterial fermentation, lactulose acidifies the intestinal lumen, enhances fecal ammonia excretion, suppresses ammonia-producing bacteria, and induces osmotic catharsis (30, 31). Probiotics, in contrast, exert their effects by modulating gut microbiota composition, reinforcing intestinal barrier function, and potentially attenuating systemic inflammation and oxidative stress (18, 32). In the present analysis, probiotics demonstrated no significant difference compared with lactulose in reducing serum ammonia levels, a finding that is biologically plausible given their complementary mechanisms targeting the gut–liver–brain axis.

Previous meta-analyses have primarily evaluated probiotics against placebo or no intervention, with relatively few studies directly comparing probiotics to active pharmacologic agents. Wibawa et al. (33) reported that probiotics were superior to placebo for MHE reversal and ammonia reduction but showed comparable efficacy to lactulose and L-ornithine L-aspartate. Another meta-analysis (34) suggested that probiotics improve MHE and reduce serum ammonia and endotoxin levels. Our results extend previous meta-analyses by focusing exclusively on direct head-to-head comparisons between probiotics and lactulose. Nevertheless, the lack of statistically significant differences should not be overinterpreted as evidence of clinical equivalence, particularly in light of limited statistical power and substantial clinical heterogeneity across trials. Accordingly, routine substitution of lactulose with probiotics cannot currently be recommended. Probiotics may represent a reasonable alternative or adjunctive option in selected patients who are intolerant of lactulose; however, this approach requires confirmation in adequately powered, rigorously designed trials.

Importantly, although the pooled RR for OHE development did not reach statistical significance, the point estimate (RR = 1.38) suggests a potential trend toward higher OHE risk with probiotics. This trend may reflect limited statistical power due to the limited number of included trials, small sample sizes, and relatively short follow-up durations, which could obscure modest differences in OHE incidence. Additionally, variability in baseline disease severity, cirrhosis etiology, and concomitant treatments across studies may have influenced OHE risk and attenuated group differences. Therefore, the observed trend underscores the need for larger, longer-term RCTs to more definitively assess the comparative impact of probiotics versus lactulose on OHE development.

Lactulose has been shown to improve neuropsychological performance and HRQL in MHE patients, preventing progression to OHE and reducing HE recurrence risk (7, 35, 36). Nevertheless, its clinical utility is often limited by gastrointestinal AEs, including abdominal distension, diarrhea, and nausea, which may impair long-term adherence. Probiotics are generally regarded as well tolerated across a range of clinical contexts (37, 38). Although our pooled analysis suggested a lower overall incidence of AEs with probiotics, this finding should be interpreted cautiously. AE reporting in the included trials was inconsistent and often limited to overall event counts without detailed descriptions of specific AE types, severity grading, or management strategies. Consequently, we could not perform a comparative analysis of specific adverse reactions. The apparent safety advantage of probiotics should therefore be considered hypothesis-generating rather than conclusive. Future RCTs should adopt standardized AE reporting, including detailed classification, severity grading, and management measures, to enable more robust safety comparisons.

From a clinical perspective, probiotics may offer certain practical advantages, including ease of administration and patient acceptability. However, given the modest size of the evidence base and substantial clinical heterogeneity among included studies, routine substitution of lactulose with probiotics cannot yet be recommended. In specific scenarios, such as lactulose intolerance or poor adherence, probiotics may represent a reasonable alternative or adjunctive strategy, but these applications require validation in adequately powered, methodologically rigorous trials. The potential synergistic effects of combined probiotic and lactulose therapy also warrant further investigation.

An important methodological consideration is the clinical and methodological heterogeneity among the included trials. Probiotic interventions differed in strains, formulations, dosages, and treatment durations, while cirrhosis etiologies and diagnostic approaches for MHE were also variable across studies. These factors may have influenced outcome definitions and effect estimates. Therefore, the pooled results should be interpreted with caution, as they reflect average effects across clinically diverse populations and interventions. Subgroup analyses were not feasible due to insufficient data and limited statistical power. Consequently, the findings should be regarded as hypothesis-generating rather than definitive, highlighting the need for future well-designed RCTs with standardized diagnostic criteria and probiotic regimens.

Despite the insights provided by this meta-analysis, several important limitations must be acknowledged. First, the number of included studies was small and individual trials enrolled limited sample sizes, reducing statistical power and precision. Second, this study was not prospectively registered, which may increase the risk of reporting bias. Third, clinical and methodological heterogeneity was substantial, including variations in probiotic formulations, diagnostic criteria, and AE reporting, which prevented subgroup analyses and limited the reliability of safety conclusions. Fourth, the included trials were geographically restricted to India, Egypt, and China, which may limit generalizability. In addition, several practical challenges may hinder the broader clinical application of probiotics, including variable product quality, strain-specific effects, and a lack of established optimal dosing. Finally, our findings may differ from other meta-analyses because we focused strictly on head-to-head RCTs, resulting in a smaller evidence base. Taken together, these limitations underscore that the current evidence should be interpreted with caution and highlight the need for future well-designed, multicenter trials.

## 5 Conclusion

This meta-analysis found no statistically significant differences between probiotics and lactulose in MHE reversal, prevention of progression to OHE, and reduction of serum ammonia levels. These findings indicate no evidence of superiority or inferiority, rather than definitive clinical equivalence. Although probiotics were associated with fewer reported AEs, this observation should be interpreted cautiously due to heterogeneous and potentially biased AE reporting. Probiotics may represent a potential alternative or adjunctive option for selected patients intolerant to lactulose; however, further well-designed, adequately powered RCTs are required to define their comparative efficacy and safety.

## Data Availability

The original contributions presented in the study are included in the article/Supplementary material, further inquiries can be directed to the corresponding authors.
