# Supplementary material for: Comparison of probiotics to lactulose for minimal hepatic encephalopathy in patients with cirrhosis: a meta-analysis of randomized controlled trials
Source: Front Med (Lausanne). 2026 Feb 12;13:1780891. doi: 10.3389/fmed.2026.1780891 (PMC12936003; doi:10.3389/fmed.2026.1780891)
Supplement: Supplementary file 3 [file Data_Sheet_2.pdf]

Original Research Protocol

BASIC DETAILS

Title

Comparison of probiotics to lactulose for minimal hepatic encephalopathy in patients with cirrhosis: A meta-analysis of randomized controlled trials

Authors

Qiufeng He, Zhili Chen, Yang Deng, Chuangjie Mao, Yue Fan

Objectives

Minimal hepatic encephalopathy (MHE) is a reversible, early stage of hepatic encephalopathy (HE). Although probiotics have been widely investigated for MHE, direct comparative evidence versus the standard therapy (lactulose) remains limited. This meta-analysis therefore aimed to perform a quantitative evaluation of the relative efficacy and safety of probiotics versus lactulose in cirrhotic patients with MHE.

Key words

minimal hepatic encephalopathy; probiotics; lactulose; randomized controlled trials; efficacy; safety

SEARCHING AND SCREENING

Searches

Searches for published systematic reviews and meta-analyses will be undertaken in the following databases: PubMed, the Cochrane Library, Embase, Web of Science, and China Biomedical Literature Database (CBM). The initial search was conducted on May 12, 2025, and updated on August 28, 2025, to include the most recent information and relevant evidence. Relevant studies were also identified by reviewing the reference lists of previous studies. No language, publication date, or publication status restrictions were applied. The following search strategy is used for PubMed and

Embase: ("Prebiotics"[Title/Abstract] OR "Prebiotic"[Title/Abstract] OR "Prebiotics"[MeSH Terms] OR "Synbiotics"[Title/Abstract] OR "Synbiotic"[Title/Abstract] OR "Synbiotics"[MeSH Terms] OR "Probiotics"[Title/Abstract] OR "Probiotic"[Title/Abstract] OR "Probiotics"[MeSH Terms]) AND ("Lactulose"[Title/Abstract] OR "Duphalac"[Title/Abstract] OR "Normase"[Title/Abstract] OR "Amivalex"[Title/Abstract] OR "Lactulose"[MeSH Terms]) AND ("Hepatic Encephalopathy"[MeSH Terms] OR "Hepatic Encephalopathy"[Title/Abstract] OR "encephalopathies hepatic"[Title/Abstract] OR "hepatic encephalopathies"[Title/Abstract] OR "encephalopathy hepatic"[Title/Abstract] OR "portal systemic encephalopathy"[Title/Abstract] OR "portal systemic encephalopathy"[Title/Abstract] OR (("brain diseases"[MeSH Terms] OR ("brain"[All Fields] AND "diseases"[All Fields]) OR "brain diseases"[All Fields] OR "Encephalopathies"[All Fields] OR "Encephalopathy"[All Fields]) AND "Portal-Systemic"[Title/Abstract]) OR (("brain diseases"[MeSH Terms] OR ("brain"[All Fields] AND "diseases"[All Fields]) OR "brain diseases"[All Fields] OR "Encephalopathies"[All Fields] OR "Encephalopathy"[All Fields]) AND "Portal-Systemic"[Title/Abstract]) OR (("brain diseases"[MeSH Terms] OR ("brain"[All Fields] AND "diseases"[All Fields]) OR "brain diseases"[All Fields] OR "Encephalopathies"[All Fields] OR "Encephalopathy"[All Fields]) AND "Portal-Systemic"[Title/Abstract]) OR "portal systemic encephalopathies"[Title/Abstract] OR "encephalopathy portosystemic"[Title/Abstract] OR "hepatocerebral encephalopathy"[Title/Abstract] OR "portosystemic encephalopathy"[Title/Abstract] OR (("brain diseases"[MeSH Terms] OR ("brain"[All Fields] AND "diseases"[All Fields]) OR "brain diseases"[All Fields] OR "Encephalopathies"[All Fields] OR "Encephalopathy"[All Fields]) AND "Portosystemic"[Title/Abstract]) OR "portosystemic encephalopathies"[Title/Abstract] OR "encephalopathy hepatocerebral"[Title/Abstract] OR (("brain diseases"[MeSH Terms] OR ("brain"[All Fields] AND "diseases"[All Fields]) OR "brain diseases"[All Fields] OR "Encephalopathies"[All Fields] OR "Encephalopathy"[All Fields]) AND "Hepatocerebral"[Title/Abstract]) OR ("Hepatocerebral"[All Fields] AND

"Encephalopathies"[Title/Abstract]) OR "hepatic coma"[Title/Abstract] OR (("Coma"[MeSH Terms] OR "Coma"[All Fields] OR "Comas"[All Fields]) AND "Hepatic"[Title/Abstract]) OR "hepatic comas"[Title/Abstract] OR "hepatic stupor"[Title/Abstract] OR (("Stupor"[MeSH Terms] OR "Stupor"[All Fields] OR "stuporous"[All Fields] OR "lethargy"[MeSH Terms] OR "lethargy"[All Fields]) AND "Hepatic"[Title/Abstract]) OR (("liver failure, acute"[MeSH Terms] OR ("liver"[All Fields] AND "failure"[All Fields] AND "acute"[All Fields]) OR "acute liver failure"[All Fields] OR ("fulminant"[All Fields] AND "Hepatic"[All Fields] AND "failure"[All Fields]) OR "fulminant hepatic failure"[All Fields]) AND "cerebral edema"[Title/Abstract]))

Study design

Randomized Controlled Trials (RCTs) comparing probiotics with lactulose in the treatment of MHE among patients with cirrhosis were included. Case reports, retrospective studies, and non-randomized clinical trials were excluded. No language restrictions. There will be no language limitation. Only human studies with full text available will be analyzed.

ELIGIBILITY CRITERIA

Condition or domain being studied

minimal hepatic encephalopathy

Population

Inclusion: cirrhotic patients age ≥18 years diagnosed with MHE, irrespective of gender or race, were included.

Exclusion: animals, pediatric participants (age <18 years), patients with overt hepatic encephalopathy (OHE).

Intervention(s) or exposure(s)

Probiotics (including synbiotics and prebiotics) for the treatment of MHE in cirrhotic patients.

No restriction on duration and dose (if applicable) of treatment will be imposed.

Comparator(s) or control(s)

Lactulose for the treatment of MHE in cirrhotic patients.

No restriction on duration and dose (if applicable) of treatment will be imposed.

Main outcomes

Reversal of MHE: defined as the improvement in any 2 neuropsychological tests; or score of psychometric hepatic encephalopathy decreased 4 points and above.

Additional outcomes

The secondary outcomes include:

1. Development of OHE: defined as a progression of grade II HE or more.

2. Reduction in serum ammonia levels: change of serum ammonia after treatment.

3. Incidence of AEs: if described serious AEs, will be reported separately.

DATA COLLECTION PROCESS

Data extraction

Two investigators independently screened the titles and abstracts of identified records against the pre-defined eligibility criteria. The full texts of all potentially relevant citations, as well as those where eligibility was uncertain, were retrieved for further assessment. A custom data extraction form was developed in Microsoft Excel to systematically capture all required data. Any disagreements during the screening or extraction process were resolved through discussion or, if necessary, by consultation with a third author.

For each included study, information will be extracted on: publication year, country, sample size, age, sex, interventions of treatment (dosage, duration), MHE diagnostic tests, Child–Pugh classification or Model for End-Stage Liver Disease. Additional information was requested from corresponding authors via email if the data were insufficient or unclear.

Risk of bias (quality) assessment

The overall quality of evidence will be evaluated by two independent authors using the Modified Jadad Scale. This tool evaluates four key domains of potential bias: random sequence generation, allocation concealment, blinding, and the handling of dropouts and withdrawals. The risk of bias for each domain was judged as 'low,' 'high,' or

'unclear.' An overall quality score (ranging from 0 to 7) was assigned to each study based on this appraisal, with a score of ≥4 indicating high quality.

PLANNED DATA SYNTHESIS

Strategy for data synthesis

Data synthesis and analysis were carried out in Stata 12.0. For outcome synthesis, dichotomous variables are reported as pooled risk ratios (RR), and continuous variables as standardized mean differences (SMD), both with 95% CIs. Statistical heterogeneity across studies was evaluated using the I^2 statistic and the Cochran Q test, with $I^2 > 50\%$ and a Q-test P-value < 0.05 considered indicative of significant heterogeneity. The choice of meta-analytic model was guided by the heterogeneity results: a fixed-effect model was applied when heterogeneity was not significant, and a random-effects model was adopted otherwise. Potential publication bias was assessed through visual examination of funnel plots for symmetry and confirmed with Egger's formal statistical test, where P > 0.05 was interpreted as no significant bias. A two-tailed P-value of less than 0.05 defined statistical significance for all hypothesis tests.

Analysis of subgroups

Subgroup analyses were conducted based on the following criteria to account for heterogeneity or attempt to analyze whether effect sizes were significantly different among subgroups (provided there were sufficient data).

1. Type of probiotics
2. Treatment duration
3. MHE diagnostic tests

AFFILIATION AND FUNDING

Review team members

Qiufeng He, Department of Hepatology, Public Health Clinical Center of Chengdu

Zhili Chen, Department of Hepatology, Public Health Clinical Center of Chengdu

Yang Deng, Department of Hepatology, Public Health Clinical Center of Chengdu

Chuangjie Mao, Department of Hepatology, Public Health Clinical Center of Chengdu

Yue Fan, Department of Blood Transfusion, Deyang People's Hospital

Funding

Not applicable.

Contact

Yue Fan

Department of Blood Transfusion, Deyang People's Hospital，No.173 Taishan North Road, Jingyang District, Deyang 618000, Sichuan, China

E-mail: 912625958@qq.com
