# Supplementary material for: Comparison of probiotics to lactulose for minimal hepatic encephalopathy in patients with cirrhosis: a meta-analysis of randomized controlled trials
Source: Front Med (Lausanne). 2026 Feb 12;13:1780891. doi: 10.3389/fmed.2026.1780891 (PMC12936003; doi:10.3389/fmed.2026.1780891)
Supplement: Supplementary file 4 [file Data_Sheet_3.pdf]

Search Strategy

PubMed

("Prebiotics"[Title/Abstract] OR "Prebiotic"[Title/Abstract] OR "Prebiotics"[MeSH Terms] OR "Synbiotics"[Title/Abstract] OR "Synbiotic"[Title/Abstract] OR "Synbiotics"[MeSH Terms] OR "Probiotics"[Title/Abstract] OR "Probiotic"[Title/Abstract] OR "Probiotics"[MeSH Terms]) AND ("Lactulose"[Title/Abstract] OR "Duphalac"[Title/Abstract] OR "Normase"[Title/Abstract] OR "Amivalex"[Title/Abstract] OR "Lactulose"[MeSH Terms]) AND ("Hepatic Encephalopathy"[MeSH Terms] OR "hepatic encephalopathy"[Title/Abstract] OR "minimal hepatic encephalopathy"[Title/Abstract] OR "portal systemic encephalopathy"[Title/Abstract] OR "portosystemic encephalopathy"[Title/Abstract] OR "hepatocerebral encephalopathy"[Title/Abstract] OR "hepatic coma"[Title/Abstract])

The Cochrane Library

(MeSH descriptor: [Probiotics] explode all trees OR probiotic*:ti,ab,kw OR prebiotic*:ti,ab,kw OR synbiotic*:ti,ab,kw) AND (MeSH descriptor: [Lactulose] explode all trees OR lactulose:ti,ab,kw OR duphalac:ti,ab,kw OR normase:ti,ab,kw OR amivalex:ti,ab,kw) AND (MeSH descriptor: [Hepatic Encephalopathy] explode all trees OR "hepatic encephalopathy":ti,ab,kw OR "minimal hepatic encephalopathy":ti,ab,kw OR "portal systemic encephalopathy":ti,ab,kw OR "portosystemic encephalopathy":ti,ab,kw OR "hepatocerebral encephalopathy":ti,ab,kw OR "hepatic coma":ti,ab,kw)

Embase

('probiotic'/exp OR probiotic*:ti,ab OR 'prebiotic'/exp OR prebiotic*:ti,ab OR 'synbiotic'/exp OR synbiotic*:ti,ab) AND ('lactulose'/exp OR lactulose:ti,ab OR duphalac:ti,ab OR normase:ti,ab OR amivalex:ti,ab) AND ('hepatic encephalopathy'/exp OR 'hepatic encephalopathy':ti,ab OR 'minimal hepatic encephalopathy':ti,ab OR 'portal systemic encephalopathy':ti,ab OR 'portosystemic

encephalopathy':ti,ab OR 'hepatocerebral encephalopathy':ti,ab OR 'hepatic coma':ti,ab)

Web of Science

TS=(probiotic* OR prebiotic* OR synbiotic*) AND TS=(lactulose OR duphalac OR normase OR amivalex) AND TS=("hepatic encephalopathy" OR "minimal hepatic encephalopathy" OR "portal systemic encephalopathy" OR "portosystemic encephalopathy" OR "hepatocerebral encephalopathy" OR "hepatic coma")

China Biomedical Literature Database (CBM)

(益生菌 OR 益生元 OR 合生元) AND (乳果糖 OR Duphalac OR Normase)
AND (肝性脑病 OR 轻微肝性脑病 OR 门体分流性脑病)
