# Supplementary material for: Comparison of probiotics to lactulose for minimal hepatic encephalopathy in patients with cirrhosis: a meta-analysis of randomized controlled trials
Source: Front Med (Lausanne). 2026 Feb 12;13:1780891. doi: 10.3389/fmed.2026.1780891 (PMC12936003; doi:10.3389/fmed.2026.1780891)

Figure S2: Sensitivity analysis by fixed-effects model of MHE reversal

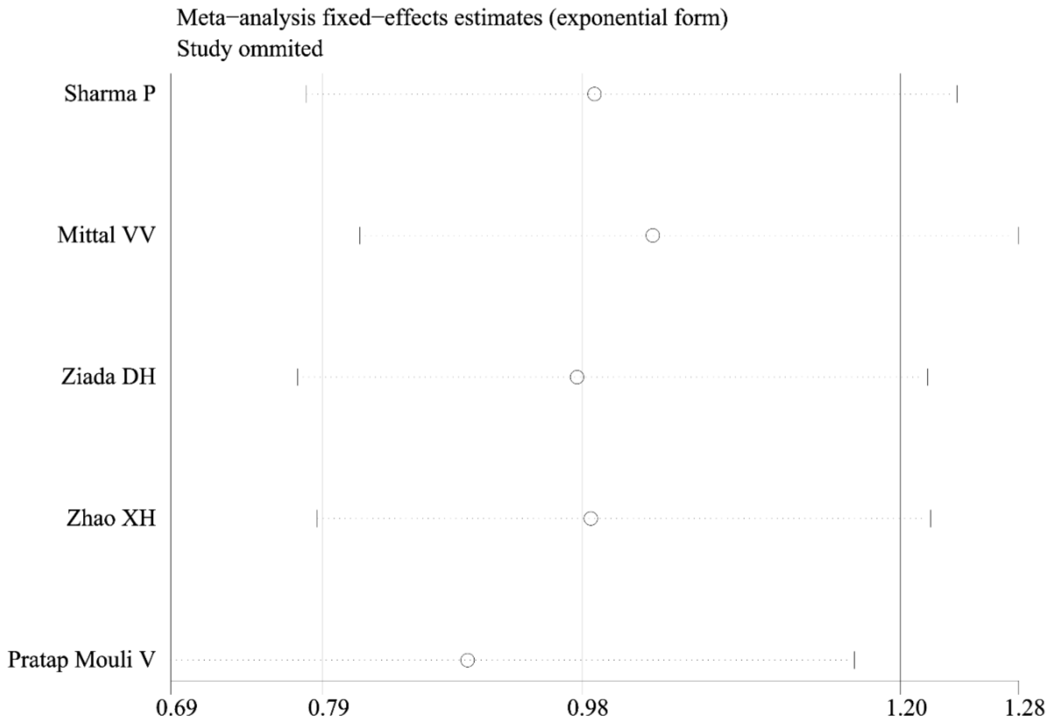

Figure S3: Sensitivity analysis by fixed-effects model of OHE development.

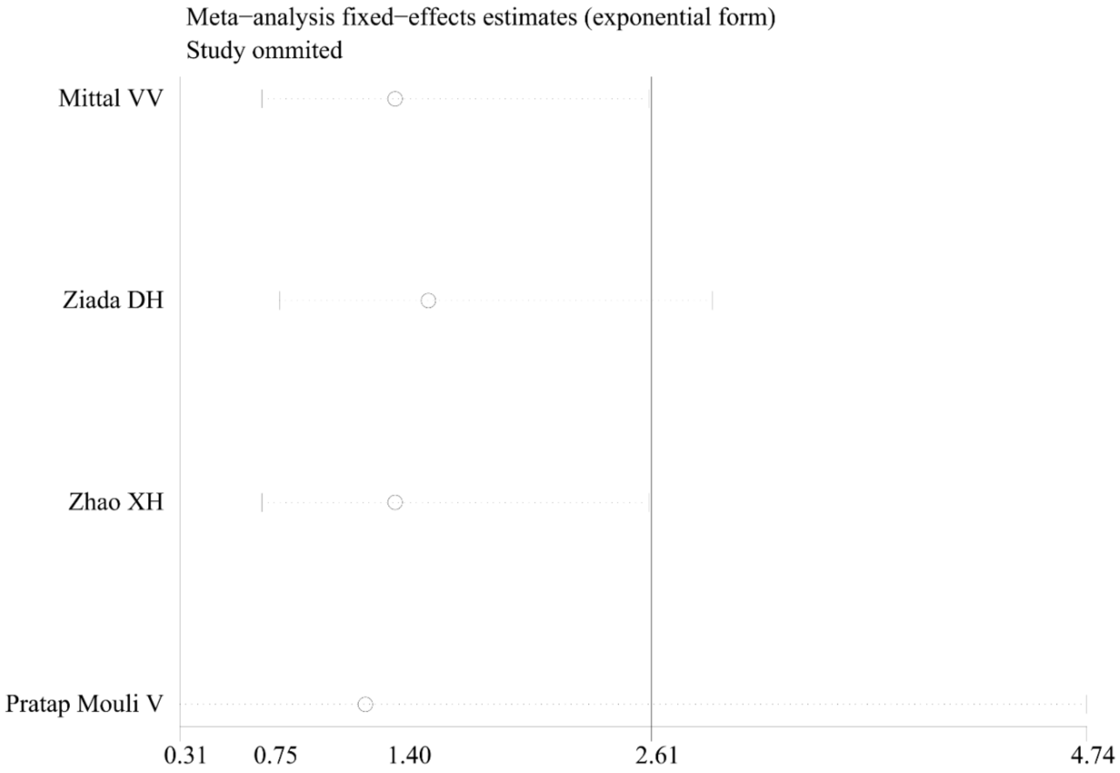

Figure S4: Sensitivity analysis by fixed-effects model of ammonia reduction.

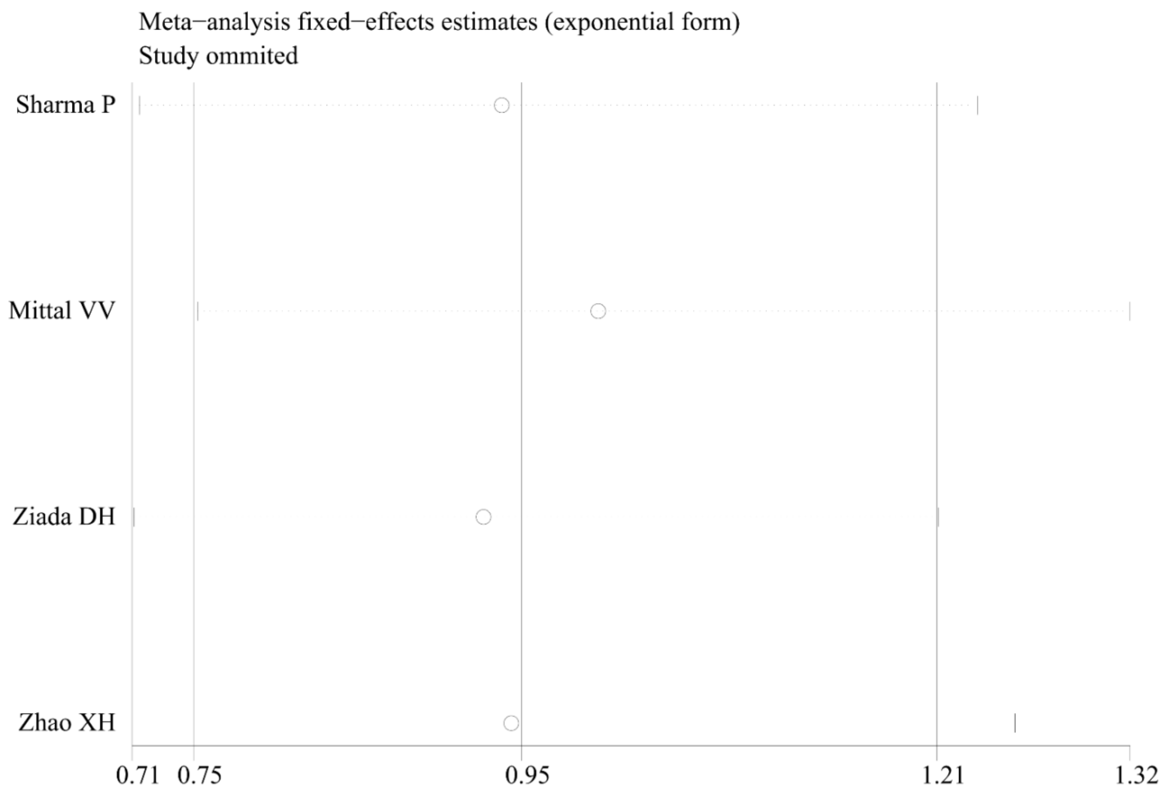

Figure S5: Sensitivity analysis by fixed-effects model of AEs.

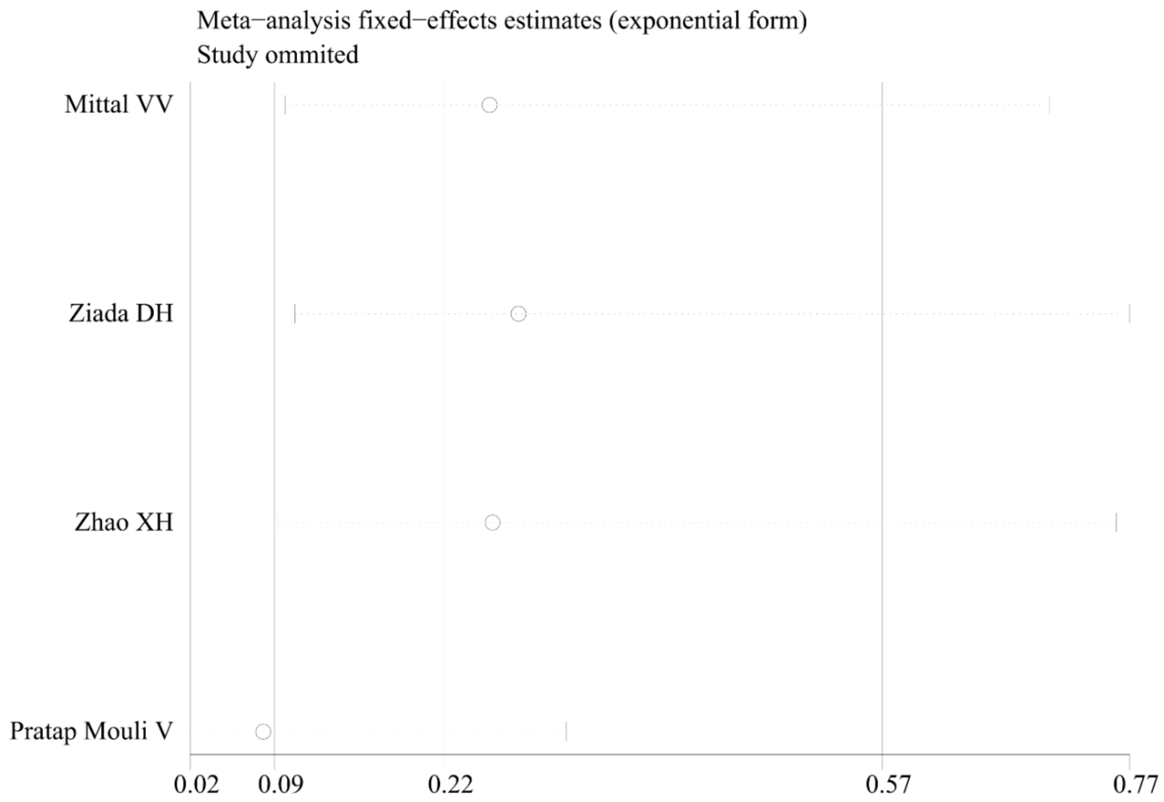

Supplement: Supplementary file 5 [file Data_Sheet_4.pdf]
